# Progress in Surgery

**Published:** 1896-02-01

**Authors:** 


### Progress in Surgery.

### CEREBRAL SURGERY.

**Distension of Frontal Sinus.**—Mr. Simeon Snell,[1] of Sheffield, contributes a paper on this subject apropos of two cases on which he has recently operated. As the frontal sinuses are not developed till the fifth or sixth year, the condition is not met with in young children. In adults, however, distension from mucus (mucocoele) or pus (empyæma) forms a distinct swelling in the upper and inner orbital angle. The swelling is accompanied by a varying amount of pain, some displacement of the eyeball in a downward and outward direction, with consequent diplopia. Both Mr. Snell's recent cases were examples of empyæma, occurring in males. In the first case, which presented all the clinical phenomena above mentioned, there was a history of an antecedent facial erysipelas of a very severe character, as well as of a direct blow on the spot at which the swelling subsequently appeared. The operation consisted in exposing and opening the sinus, clearing it out after scraping, and establishing a drain into the nose. This was maintained for six or seven weeks, and the patient made a complete recovery, the eyeball resuming its natural position, and the diplopia disappearing. In the second case no cause was assigned, and after increasing in size for about a year, the swelling burst into the eyelid, and a discharging sinus persisted for some time. The frontal sinus was freely opened and drained into the nose. The treatment was more prolonged and complicated than in the first case, doubtless due to the spontaneous opening of the abscess.

The condition has to be diagnosed from distension of the lachrymal sac and tumour of the inner wall of the orbit, and, as a rule, there is little difficulty in doing so. Bilateral distension is rare. In treatment it is most important to re-establish the communication with the nose and to retain a drain in for a considerable time. The author specially mentions drowsiness as a symptom of this condition, a point which Mr. Pye-Smith has also observed.

**Mastoid Disease.**—In a paper on the various methods of operating on the mastoid process, Dr. A. Bronner,[2]

of Bradford, expresses his preference for Stacke's operation as modified by Macewen over others. He draws particular attention to (1) the importance of an accurate knowledge of the anatomy and pathology of the parts before undertaking operations in this region; (2) the advisability of exploring the mastoid in cases of chronic otitis media which resist ordinary treatment; (3) the danger and limited value of the gimlet or gouge alone in the operation; (4) the great importance of obtaining healing from the bottom by plugging with gauze and using no stitches in the wound.

**Primary Thrombosis of Superior Longitudinal Sinus.**—Dr. E. Hugh Snell[3] reports a case of a young woman who suffered from severe headache, with loss of power of the right arm and leg, and inability to stand or walk. The illness began with vomiting and diarrhœa, accompanied by severe headache; followed by paralysis of right arm and leg and convulsive fits on the same side. Among other symptoms were impairment of the intellect, tremulous tongue, and optic neuritis. No œdema of the scalp or forehead was present. Under tonics, stimulants, and good diet the patient made a gradual recovery.

**Excision of Cortical Areas in Idiopathic Epilepsy.**—Eulenburg[4] reports a case of idiopathic epilepsy relieved by excision of a portion of the motor cortex. While the results of such operations have not been very satisfactory, he thinks the operation entirely rational and worthy of a wider trial, especially in cases in which no definite pathological condition exists, and a purely functional alteration of the cortex is present. In his own case there was immunity from fits for seven months after excision of the right arm centre.

[1] Quart. Med. Jour., Oct., 1895. [2] Lancet, Nov. 9, 1895. [3] Ibid. [4] Berliner Klinische Woch., 15, 1895.

